# Stressful Life Memories Relate to Ruminative Thoughts in Women With Sexual Violence History, Irrespective of PTSD

**DOI:** 10.3389/fpsyt.2018.00311

**Published:** 2018-09-05

**Authors:** Emma M. Millon, Han Yan M. Chang, Tracey J. Shors

**Affiliations:** Behavioral and Systems Neuroscience, Department of Psychology, Center for Collaborative Neuroscience, Rutgers University, Piscataway, NJ, United States

**Keywords:** sexual violence, stress, trauma, PTSD, depression, rumination, memory, fear

## Abstract

More than one in every four women in the world experience sexual violence (SV) in their lifetime, most often as teenagers and young adults. These traumatic experiences leave memories in the brain, which are difficult if not impossible to forget. We asked whether women with SV history experience stronger memories of their most stressful life event than women without SV history and if so, whether strength relates to ruminative and trauma-related thoughts. Using the Autobiographical Memory Questionnaire (AMQ), women with SV history (*n* = 64) reported this memory as especially strong (*p* < 0.001), remembering more sensory and contextual details, compared to women without SV history (*n* = 119). They further considered the event a significant part of their personal life story. The strength of the memory was highly correlated with posttraumatic cognitions and ruminative thoughts, as well as symptoms of depression and anxiety (*p's* < 0.001, *n* = 183). A third (33%) of the women with SV history were diagnosed with posttraumatic stress disorder (PTSD), but PTSD alone did not account for the increase in memory strength (*p*'s < 0.001). These data suggest that the experience of SV increases the strength of stressful autobiographical memories, which are then reexperienced in everyday life during posttraumatic and ruminative thoughts. We propose that the repeated rehearsal of vivid stressful life memories generates more trauma memories in the brain, making the experience of SV even more difficult to forget.

## Introduction

Sexual violence (SV) against women is common in today's world with numbers upward of 25% (1, 2). Most experiences occur during adolescence and young adulthood, when women are most vulnerable (3). The numbers are also high for women in college. Several years ago, the White House Task Force partnered with the Bureau of Justice to survey SV on nine college campuses. Of nearly 15,000 responders, ~10% reported SV during college (4). Other surveys suggest numbers closer to 25% (5, 6). Percentages are even higher (32%) for women in the same age group but not enrolled in college (6).

Memories of an extremely stressful life event tend to be easily recalled and are generally stronger than memories of normal-day experience (7–9). They also change with time as the memory is rehearsed and/or avoided (10). When a memory is reactivated, it becomes associated with the new context (11). If the context is safe and/or neutral, the strength of the memory may lessen and its expression can extinguish. This process of “extinction” forms the basis of exposure therapy, the most accepted evidence-based intervention for people with trauma history (12). The memory is not erased from the brain but exposure therapy is effective because cues associated with the event are less likely to elicit the conditioned fear response. However, others theorize that rehearsing the trauma memory can strengthen the memory simply because it is being reactivated and reconsolidated over and over again. Because of these concerns, it is important to understand more fully how someone relives the memory of an extremely stressful life event, such as sexual violence. Does the person remember when and where the event took place more or less than the feelings associated with the event? Does he or she tend to remember discrete sensory details such as the sounds and smells or rather is the memory relived like a movie in sequence? Does he or she feel as if traveling back in time or is the memory experienced in the present moment?

Posttraumatic Stress Disorder (PTSD) is a mental illness characterized by persistent trauma-related thoughts after an extremely stressful life event (13). Most individuals who experience SV do not go on to develop PTSD but are at high risk. In fact, of all traumas, rape is the most likely to induce PTSD (13, 14). A diagnosis includes some combination of intrusive thoughts, flashbacks, avoidance behaviors, numbness or hyperarousal, along with disruption of normal life function (15). Although many people with PTSD suffer from symptoms of depression and anxiety (11, 16), trauma memories are the source of most problems (17, 18). According to Rubin and colleagues, “PTSD is defined in large part by changes that occur specifically in autobiographical memory” (19). To assess the strength of these memories, they developed the Autobiographical Memory Questionnaire (AMQ), which asks questions related to the details of an autobiographical memory, typically framed as a negative event in their past. In response to the AMQ, individuals with PTSD recalled negative autobiographical events with greater strength compared to people without PTSD, and the strength of the memory correlated with PTSD symptoms (9, 19–21). They interpret these and other findings to suggest that the repeated rehearsal of an intense memory experience may exacerbate PTSD symptoms over time and contribute to one's life story (19). To our knowledge, the AMQ has not been used to assess the strength of stressful life memories in women who experienced the trauma of SV while adolescents and young adults, as it relates to the diagnosis of PTSD.

Rumination is defined as the repeated rehearsal of thoughts. These thoughts are usually autobiographical, negative in nature and about the past. They are also described as uncontrollable and involuntary. Historically, rumination was considered a trait and thus relatively stable. As such, the tendency to ruminate was considered a potential risk factor for PTSD (22, 23). However, more recent data suggest that rumination is malleable and can be decreased significantly by interventions, which target trauma memories (24–27). In general, ruminative thoughts have been most often associated with depression, although recent studies, including our own, indicate a strong relationship to trauma (22, 28). But exactly *how* they relate to trauma is unclear. Minimally, they exacerbate trauma-related thoughts through the repetition of trauma memories (28) and as discussed, each time a memory is retrieved, a new memory is made through its association with the context in which it is expressed. Thus, at a neuroscience level, one could hypothesize that the repetitive and largely involuntary rehearsal of a trauma memory creates yet more memories of the trauma and related memories in the brain. A related theory, known as the “multiple trace theory” was developed by Nadel and Moscovitch to account for the persistence of memories (29–31). We extend their theory to suggest that the repeated rehearsal of a vivid autobiographical memory during rumination generates more trauma memories, thereby making the trauma more difficult to “forget.” The purpose of the present study was to investigate the relationship between rumination and the strength of autobiographical memories in women with SV history.

In the present study, we hypothesized that women with SV history would report autobiographical memories of a stressful life event as more intense than women without SV history, even in the absence of PTSD. It was hypothesized that women who ruminate more would also report stronger stressful life memories. We further hypothesized that the experience of SV history rather than the diagnosis of PTSD *per se*, would influence the expression of ruminative thoughts and intensity of stressful memories. Finally, we hypothesized that the relationship between the stressful life memory and rumination would relate to the numbers of trauma-related cognitions, as well as anxious and depressive symptoms. Adult women who experienced SV during and after puberty were evaluated because most women are assaulted during this time period and because memory processes for events that occur in childhood may be different from those acquired later (3). To test the hypotheses, we relied on statistical analyses of group differences between women with and without SV history as well as correlations among outcomes within individuals.

## Methods

### Participants and procedure

One hundred and eighty three college-aged women (*M*_age_ = 20 years, *SD* = 2.67, *range* 18–39 years; 33.2% Asian, 30.4% European/Caucasian/White; 16.8% African-American/Black/Caribbean; 9.8% Hispanic/Latina; 9.8% more than one race/other/unknown) participated in testing at a northeastern university. Participants were included if they were able and willing to provide written informed consent and excluded if they were over 40 years of age. Less than 10% of the participants were currently prescribed anti-anxiety medications or anti-depressants. Sixty four participants (*n* = 64) reported experiencing sexual violence, and one hundred nineteen participants (*n* = 119) did not experience SV and served as controls.

This study was carried out in accordance with the recommendations of the Institutional Review Board at Rutgers University. All participants provided written informed consent in accordance with the Declaration of Helsinki. Participants were assessed for trauma history with a structured clinical interview, completed a series of questionnaires (described below) and then completed the working memory task (total session ~2 h). Afterwards, participants were debriefed and compensated ($20 or research credits).

### Materials

All participants were assessed for trauma history with the Structured Clinical Interview for DSM-5 (SCID; 15) by a doctoral psychology graduate student trained in conducting clinical interviews. Trauma exposure was defined according to the DSM-5 as direct exposure to “an event or events that involved actual or threatened death, serious injury or sexual violation to the self” (32). The age of participant at time of trauma was also recorded and used to select only those participants with trauma history during adolescence or young adulthood for analyses.

The *Autobiographical Memory Questionnaire* [AMQ; (33)] is a 19-item questionnaire that assesses qualities of an autobiographical memory. Given the breadth of the questionnaire, typically items are scored individually or in clusters, depending on the research question of interest (20). We were especially interested in the sensory details and vividness of details related to the memory rather than the accuracy of the memory itself. Therefore, we did not assess items concerning confidence of the memory or dating the event. We also wanted to protect their privacy and therefore, did not ask them to identify the event. Participants were told to respond to the questionnaire in relation to an autobiographical memory of *the most stressful event in your life*. We included 12 individual items which assessed the rehearsal of the memory, sensory details (“*As I remember the event, I can hear it in my mind*” or “*see it in my mind*”), temporal and spatial details (“*As I remember the event, I know its temporal and spatial layout*”), emotional intensity (“*As I remember the event, I feel the emotions now that I felt then”*), and significance (“*the memory is significant in my life*”). Total scores were calculated, with greater scores representing heightened vividness of details related to a stressful autobiographical memory. This task has been used in the past to distinguish between types of memory systems activated during recall, particularly in participants with PTSD symptoms (7, 19, 20, 34).

The *Symmetry Span Task* was used to assess working memory (35). Developed by Engle and colleagues, the computer task required participants to make assessments of symmetrical pictures while remembering the temporal order and spatial location of a series of squares individually displayed on a grid. Participants were shown a picture and were asked to respond whether it was symmetrical or not. Immediately following, a 1 × 1 square in a 4 × 4 grid appeared on the screen for 2-s. Pairs of pictures and squares were presented 2–5 times per trial, for 12 trials (3 blocks total). Following stimulus presentations, participants were asked to replicate the location of the squares in the order presented. The task required ~20 min to complete. Data from the working memory task yielded partial and absolute accuracy scores. A partial accuracy score was a sum of all correct responses. An absolute accuracy score was the sum of correct responses in completely correct trials, when the order and location of all squares were correctly identified during the trial (all-or-none system).

The *Ruminative Responses Scale* [RRS; (36)] is a 22-item questionnaire that assesses thoughts and responses to depressed mood and affect. Examples of RRS items include “*thinking about how sad you feel*,” “*thinking about your shortcomings, failings, and mistakes*” or “*analyzing events to understand why you feel depressed or unmotivated*.” The RRS is scored as a summation of responses (min 22; max 88) as well as according to three subscales: (1) depressive ruminations, which relate to the rehearsal of depressive events, (2) brooding ruminations, which are often non-adaptive and emotion-laden, and (3) reflective ruminations, which are not as maladaptive but self-focused (37, 38).

The *Posttraumatic Cognitions Inventory* (PTCI) is a 33-item questionnaire that assesses altered cognitions related to traumatic life experiences (16). The PTCI is composed of three subscales: thoughts related to oneself (i.e., sees oneself as blameworthy, isolated, unreliable), others (i.e., sees them as untrustworthy), and the world (i.e., sees it as a dangerous place). The purpose of the PTCI is to assess the person's reaction to the event after time has passed and includes items such as “*I have to be on guard all the time*,” “*I feel like I don't know myself anymore*,” and “*I feel isolated*.” We altered the prompt to ask participants about their thoughts and feelings related to *the most stressful event of your life*. This way, women with no trauma history could also report their thoughts and feelings of a past event. We also wanted to use the same prompt as used for the AMQ. Higher PTCI scores are indicative of more negative posttraumatic cognitions and are generally associated with greater numbers of PTSD symptoms (24).

The *Beck Depression Inventory* [BDI; (39)] is a 21-item questionnaire that measures symptoms of depression. The *Beck Anxiety Inventory* [BAI; (40)] is a 21-item questionnaire that measures symptoms of anxiety. For both measures, total scores are computed by adding response choices. Scores greater than 20 on the BDI represent moderate to severe depression and scores greater than 20 on the BAI are consistent with moderate to severe anxiety. Both questionnaires are commonly used to assess cognitions associated with symptoms of depression and anxiety, respectively (41–43).

### Statistical analyses

Dependent variables were obtained from self-report scores from the respective questionnaires with independent variables based on violence, PTSD, or trauma history. Independent samples *t*-tests and analyses of variance (ANOVA) were used to test differences in mental health outcomes (AMQ, Working Memory task, RRS, PTCI, BDI, BAI) between groups without (no-SV, *n* = 119) and with history of SV (SV, *n* = 64). The sample was divided further to account for PTSD diagnosis: no-SV, SV-no PTSD (*n* = 43), SV+PTSD (*n* = 21). When analyzing groups based on SV history and PTSD diagnosis, ANOVAs and Fisher's least significance difference (LSD) *post-hoc* test were used to uncover differences in the strength of autobiographical memories and ruminative thoughts between groups.

Relationships among all outcome measures are presented as Pearson *rho* correlations. Statistical analyses were assessed with SPSS version 24.0 (IBM Corp., 2017) and a significance level of *p* < 0.05 was used for all analyses, with Bonferroni corrections for the AMQ. Effect sizes are presented as partial eta-squared (η^2^) in order to quantify meaningful differences in dependent outcomes between women with and without SV history. Typically, partial η^2^ effect sizes are categorized as 0.01 (small), 0.09 (medium), and 0.25 (large) (44).

## Results

### Sexual violence history with and without PTSD

Approximately one-third of women with SV history (*n* = 21) met diagnostic criteria for current PTSD according to SCID-5 criteria. Women with SV history regardless of PTSD diagnosis averaged seven current PTSD symptoms (*SD* = 5, *range* 0–19). Women with exposure to trauma other than SV (*n* = 19) averaged one current PTSD symptom.

### Autobiographical memory

**Figure 1:**
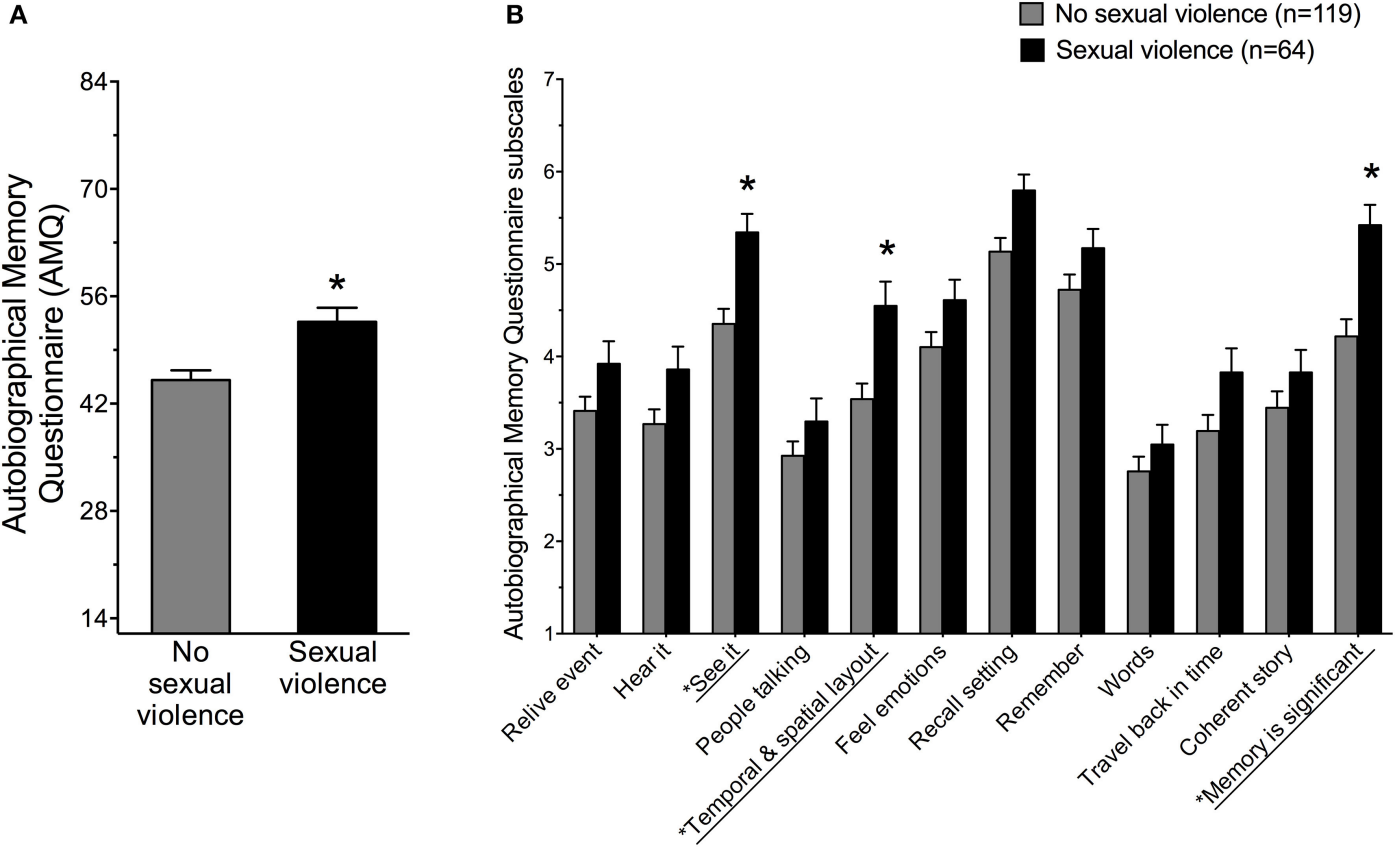
**(A)** Women with sexual violence history reported significantly more details of an autobiographical memory of a past stressful event compared to women with no sexual violence history as assessed by the Autobiographical Memory Questionnaire (AMQ) ^*^p < 0.001. **(B)** Women with sexual violence history reported significantly more details related to seeing the event in their mind, the temporal and spatial layout of the memory as well as the significance the memory played in their life compared to women with no sexual violence history. ^*^p < 0.005, with Bonferroni correction for multiple comparisons.

The cumulative AMQ score from items 1–12 was calculated for women with SV history vs. women without SV history. Women with SV history reported a significantly stronger memory of their most stressful life event compared to women with no history of SV *F*_(1, 181)_ = 11.75, *p* < 0.001, Partial η^2^ = 0.06 (Figure 1A). Women with SV and PTSD did not report higher AMQ scores than women with SV but no PTSD, *F*_(2, 180)_ = 6.53, *p* > 0.05, but they did report higher AMQ scores than no-trauma controls. Women with SV history but no PTSD reported stronger AMQ memory scores compared to women without SV history (*p* < 0.05).

Items on the AMQ were analyzed for group differences. To correct for multiple comparisons and avoid Type 1 Errors, Bonferroni corrections were applied to a *p*-value of 0.05 (0.05/11 comparisons of 12 AMQ items = 0.005). Therefore, a *p*-value less than 0.005 was necessary to establish significance. Group scores were significantly different for three items (Figure 1B). Women with SV history (1) reported more details related to seeing the event in their mind, *t*_(181)_ = −3.73, *p* < 0.001, (2) indicated more details related to the spatial layout of the memory *t*_(181)_ = −3.35, *p* < 0.001, and (3) reported the autobiographical memory as significantly more central to their life story, *t*_(181)_ = −4.03, *p* < 0.001. Group responses did not differ significantly on the other nine items assessed by the AMQ, *p* > 0.005, with the Bonferroni correction, although the overall AMQ score was significantly larger in women with SV history.

### Working memory

Data collected for spatial and temporal working memory with the Symmetry Span task were analyzed. Sixty five participants did not complete the task due to technical problems. Women with SV history (*n* = 48) did not significantly differ from women without SV history (*n* = 76), on either partial accuracy, *t*_(122)_ = 1.26, *p* > 0.05, or absolute accuracy, *t*_(119.93)_ = 1.29, *p* > 0.05.

### Ruminative thoughts

**Figure 2:**
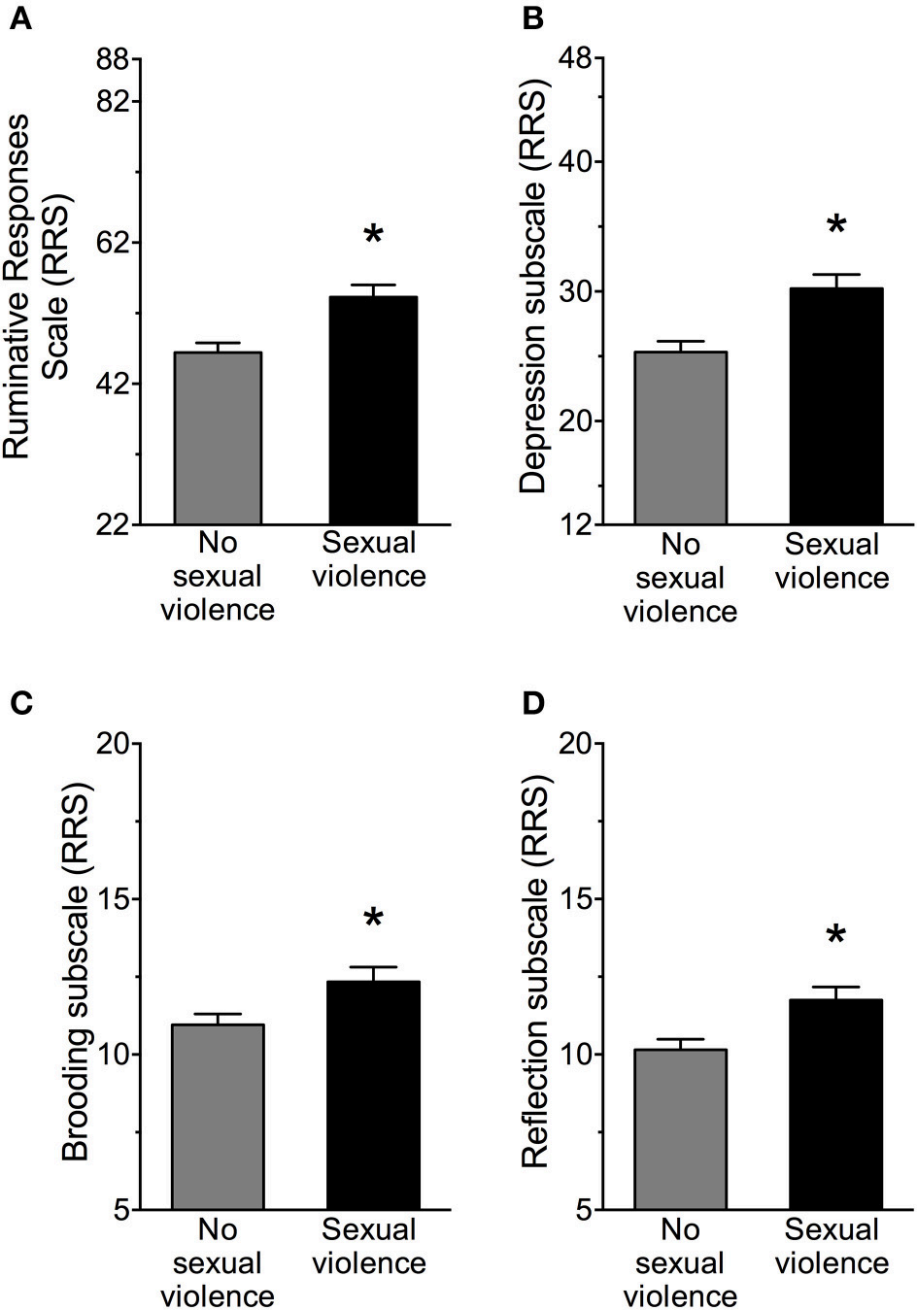
**(A)** Women with sexual violence history reported significantly greater numbers of ruminative thoughts as assessed by the Ruminative Responses Scale (RRS), as well as more **(B)** depressive, **(C)** brooding, and **(D)** reflective subtypes compared to women with no sexual violence history. ^*^p < 0.001.

Women with SV history reported ~15% more rumination as assessed with the Ruminative Responses Scale (RRS) compared to women without SV history, *F*_(1, 181)_ = 12.15, *p* < 0.001, Partial η^2^ = 0.06 (Figure 2A). RRS subscales for the depressive, brooding and reflective thoughts were analyzed (Figures 2B–D). Women with SV history reported 17% more depressive rumination compared to women without SV history, *F*_(1, 181)_ = 12.51, *p* < 0.001. Women with SV history reported 11% more brooding compared to women without SV history, *F*_(1, 181)_ = 5.54, *p* < 0.001. Additionally, women with SV history also reported 14% more reflection compared to women without SV history, *F*_(1, 181)_ = 8.32, *p* < 0.001. Women significantly differed on RRS scores depending on PTSD diagnosis, *F*_(2, 180)_ = 8.46, *p* < 0.001, Partial η^2^ = 0.09. Fisher's LSD *post-hoc* test revealed that women with SV and PTSD reported more rumination than women with SV but no PTSD, *p* < 0.05. Women with SV and no PTSD reported more rumination than women without SV, *p* < 0.05.

### Trauma-related thoughts, depression and anxiety

Women with SV history reported 26% more posttraumatic cognitions as assessed on the PTCI compared to women without SV history, *F*_(1, 181)_ = 38.42, *p* < 0.001, Partial η^2^ = 0.18 (Figure **4A**). PTCI subscales of self, world and others were analyzed. Women with SV history significantly differed from the no-SV group on all three PTCI subscales of self-blame, negative thoughts about the world and of others (*p*'s < 0.01). Women significantly differed on PTCI scores depending on PTSD diagnosis, *F*_(2, 180)_ = 24.87, *p* < 0.001, Partial η^2^ = 0.22. Fisher's LSD *post-hoc* test revealed that women with SV and PTSD reported more posttraumatic cognitions than women with SV but no PTSD, *p* < 0.05. Women with SV and no PTSD reported more posttraumatic cognitions than women without SV, *p* < 0.01.

Women with SV history reported 44% more depressive symptoms as scored on the BDI compared to women without SV history, *F*_(1, 181)_ = 25.66, *p* < 0.001, Partial η^2^ = 0.12 (equal variances not assumed) (Figure **4B**). Women significantly differed on BDI scores depending on PTSD diagnosis, *F*_(2, 180)_ = 15.34, *p* < 0.001, Partial η^2^ = 0.15. Fisher's LSD *post-hoc* test revealed that women with SV and PTSD reported over 50% more depressive symptoms than women with SV and no PTSD, as well as women without SV, *p's* < 0.05. Women with SV and no PTSD also had more depressive symptoms than women without SV, *p* = 0.001.

Women with SV history reported twice as many anxiety symptoms as assessed on the BAI compared to women without SV history, *F*_(1, 181)_ = 25.09, *p* < 0.001, Partial η^2^ = 0.12 (equal variances not assumed) (Figure **4C**). Differences in anxiety depended on PTSD diagnosis, *F*_(2, 180)_ = 16.59, *p* < 0.001, Partial η^2^ = 0.16. Fisher's LSD *post-hoc* test revealed that women with SV and PTSD reported over 70% more anxious symptoms than women with SV and no PTSD, as well as women without SV, *p's* < 0.05. Women with SV and no PTSD had more anxious symptoms than women without SV, *p* < 0.01.

### Individual differences

**Figure 3:**
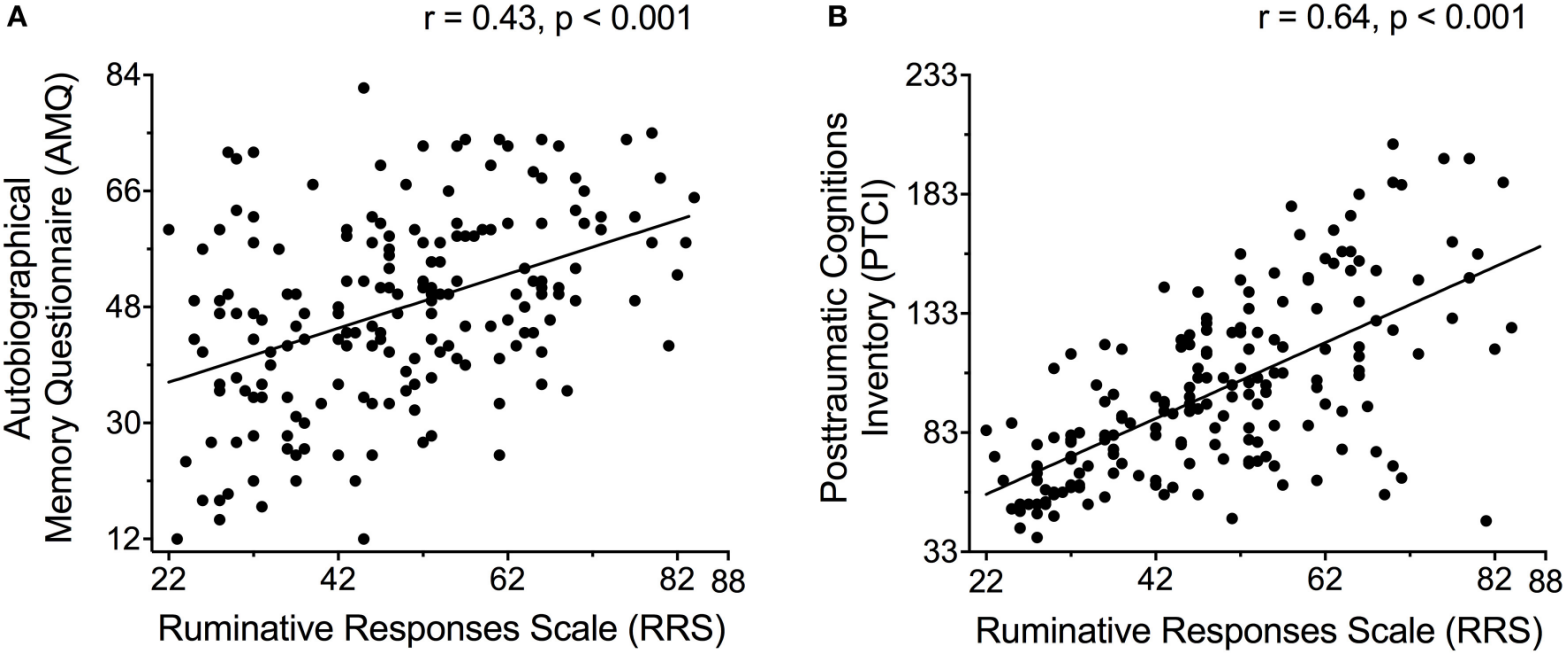
**(A)** Recall of an autobiographical memory of a past stressful event as assessed by the Autobiographical Memory Questionnaire (AMQ) and **(B)** trauma-related thoughts as assessed with the Posttraumatic Cognitions Inventory (PTCI) correlated with ruminative thoughts as assessed by the Ruminative Responses Scale (RRS).

As summarized in Table 1, women who reported more ruminative thoughts (higher RRS scores) reported a greater number of details surrounding an autobiographical memory of a past stressful event and more posttraumatic cognitions (*p* < 0.001; Figure 3). Relationships among depressive, anxious and posttraumatic symptoms across the entire sample were highly significant (*p* < 0.001).

**Table 1:** Pearson correlations coefficients among all measures.

	**AMQ**	**RRS**	**RRS-D**	**RRS-B**	**RRS-R**	**PTCI**	**BDI**
RRS	0.426^**^	—					
RRS-D	0.373^**^	0.962^**^	—				
RRS-B	0.501^**^	0.870^**^	0.775^**^	—			
RRS-R	0.279^**^	0.761^**^	0.609^**^	0.611^**^	—		
PTCI	0.400^**^	0.643^**^	0.633^**^	0.611^**^	0.395^**^	—	
BDI	0.360^**^	0.646^**^	0.665^**^	0.591^**^	0.352^**^	0.785^**^	—
BAI	0.349^**^	0.535^**^	0.517^**^	0.502^**^	0.361^**^	0.621^**^	0.758^**^

n = 183;

** p < 0.001.

*AMQ, Autobiographical Memory Questionnaire; RRS, Ruminative Responses Scale; RRS-D, RRS depressive subscale; RRS-B, RRS brooding subscale; RRS-R, RRS reflective subscale; PTCI, Posttraumatic Cognitions Inventory; BDI, Beck Depression Inventory; BAI, Beck Anxiety Inventory*.

## Discussion

In this study, we asked whether stressful life memories would be reported as more intense in women with a history of sexual trauma and if so, whether they would relate to posttraumatic cognitions and ruminative thoughts within individuals. We also asked whether these relationships would occur irrespective of a PTSD diagnosis. Researchers have long been interested in questions such as these (20, 34, 45–47). The AMQ was developed by Rubin and colleagues to answer some of these questions (33). Through a series of studies, they established traumatic memory recall as part of a basic memory system rather than its own specific system. In other words, the processes through which autobiographical memories of stressful life events are recalled may not be substantively different from the way that other memories are recalled. We used their autobiographical memory questionnaire here to compare the strength of stressful life memories in women with and without SV history and further to compare responses in women who did and did not meet criteria for current PTSD. We predicted that women with SV history would report a stressful life memory as more intense compared to responses from women without SV history and this response would not depend on the concurrent diagnosis of PTSD. As predicted, SV in the past, irrespective of PTSD diagnosis, was associated with a stronger reported memory experience. Thus, SV is sufficient to establish an especially strong autobiographical memory of a very stressful life event upon recall.

In the present study, women with SV history not only reported a stronger stressful life memory, they reported specific details as being especially prevalent (Figure 1A). They were more likely to: (1) see the event in their mind, (2) recall its temporal and spatial layout, and (3) consider the memory a significant part of their life story (Figure 1B). They did not report the “feelings” associated with the memory as more intense, nor did they report more “reliving” of the memory, when compared to women without SV history reflecting on the most stressful event in their life. These differences in memory detail suggest that the memory for a SV event is experienced as watching a movie rather than a body response, *per se*. Of course, memories for trauma are experienced in the body—the participants just did not report it as more intense. One limitation of this study is that we did not ask participants to identify the event they were reflecting upon and therefore do not necessarily know whether women were recalling the sexually violent experience. Nonetheless, these data suggest that memories for stressful life events are especially vivid in sensory and contextual detail as well as meaningful for women who have had a sexually violent experience.

Working memory is a learning process, which allows the brain to hold and manipulate information in short-term memory, usually in the service of completing a cognitive task or skill (48). Women with SV history performed a working memory task just as well as women without SV history. These findings are generally consistent with others (49). For example, college students with interpersonal trauma history performed just as well on tests of working memory and cognitive flexibility (using digit-span and card-sorting tasks) as students without trauma history (50). Another study compared the recall of autobiographical to nonautobiographical memories in individuals with PTSD. Whereas, autobiographical memories were more disorganized, nonautobiographical memories were not (51). These data and those we report here suggest that trauma and/or violence history is more likely to impact stressful life memories rather than general memory processes.

The psychological mechanisms through which SV and memory interact is not without controversy (45, 52). Many studies point to disorganization of memory (10, 51). Rape victims often describe their memories as emotionally intense but lacking coherence (53), whereas other victims report memories rich in detail. A recent study asked survivors of domestic violence to write a narrative about a traumatic event and a positive experience (54). The trauma narratives were detailed and more coherent than the memories of a positive experience, and were better predictors of PTSD. The underlying psychopathology, if present, is also important. For example, undergraduates with PTSD reported more sensory details of a traumatic event whereas undergraduates with depression recorded fewer details (55). Age also seems to matter. For example, children sometimes dissociate during trauma and as a consequence, may have difficulty remembering what happened (56). We only tested women who had experienced SV during or after puberty. Their responses suggest a strong detailed memory for their most stressful life event, which is consistent with some studies in adults (45, 57).

### Ruminations

Ruminations generate intrusive memories which can extend the persistence of PTSD (58, 59). In the present study, women with SV history reported they tend to ruminate about 15% more than women without SV history (Figure 2A). These women also reported significantly more depressive, brooding and reflective ruminations. Rumination was further associated with posttraumatic cognitions, which are indicative of PTSD symptomology. These data are consistent with meta-analyses, indicating positive relationships between rumination and posttraumatic symptoms, even years after the trauma (57, 60–63). The mechanisms through which ruminative and posttraumatic thoughts interact are unknown. In one study, individuals with PTSD described their ruminations as uncontrollable thoughts about critical life events (28). In our study, women with SV history and PTSD reported more ruminations than women with SV history and no PTSD, who in turn reported more ruminations than women without SV history. Therefore, PTSD symptoms are associated with a greater tendency to ruminate but exposure to SV on its own is sufficient to increase rumination. Of course, we cannot determine whether the tendency to ruminate predates trauma exposure and consequently exacerbates the development of trauma-related symptoms later in life, though other studies suggest this may be the case (22).

### Relations among ruminative thoughts and stressful life memories

In general, participants who reported one outcome in this study were more likely to report another outcome (Table 1). Strong correlations among responses may not be especially surprising because the questions on the various questionnaires do overlap. But even so, the correlations were strong and pervasive. For this study, we focused primarily on the relationship between rumination and stressful life memories (Figure 3). The correlation between RRS and AMQ scores was highly significant across the entire sample, as shown in Table 1; the correlation between RRS and AMQ for SV without PTSD was significant, albeit less strong (*r* = 0.33; *p* < 0.05). To our knowledge, no study to date has reported a *positive* relationship between rumination and autobiographical memory detail in women with sexual trauma exposure but not necessarily experiencing PTSD. To interpret these results, we come back to the “multiple memory trace” hypothesis. Each time a memory is activated in a new context, additional details are incorporated into the memory and a new “hippocampal trace” memory is generated (29, 30, 64–66). These learning processes in the hippocampus can interact with stress as well. In one study, people watched a stressful film and later were exposed to cues, which reactivated the memory for the film. Those who did so under stressful conditions (high cortisol) reported more intrusive memories than those who did so under unstressed conditions (67). A similar process may be occurring in women with SV history. According to their self-report surveys, they often rehearse trauma memories. The rehearsals are likely done under stressful conditions because the women are experiencing moderate levels of depression and anxiety. We propose these involuntary rehearsals generate new memories with new contextual details in the brain, which become more difficult to extinguish.

The positive relationship between rumination and memory reported here is different from other reports. Kleim and Ehlers (68) observed *less* detail for autobiographical memories in individuals with acute stress disorder (68). Moreover, the correlation between memory detail and rumination was negative. There are some key differences between studies. In theirs, memories were provoked with cues using the Autobiographical Memory Test (AMT) and thus different in content from “the most stressful life memory” retrieved during the AMQ. Also, their assessments were within weeks of the trauma and ours were generally much later. But interestingly, they conclude that an overgeneralized memory is not necessarily the result of trauma, *per se*, but rather a predisposition to depression, which arises as a result of trauma. The literature connecting trauma with autobiographical memory is extensive and implicates other factors such as avoidance behavior and executive control (17, 18, 69). Others studies propose even more complicated relationships (70–74). It is difficult to directly compare our results with these because of the methodological differences and because most of our participants did not have PTSD. In general, there does appear to be a strong relationship between rumination and the strength of autobiographical memory recall, but the direction of this relationship depends on the experimental question and procedures, as well as the presence of PTSD and/or other mental disorders such as depression.

Rumination is considered a risk factor for depression, in part because women tend to ruminate more than men do and are more often diagnosed with depression (75, 76). However, rumination *per se* does not necessarily account for the high incidence of depression in women (37). In one of our recent studies, both men and women with high numbers of depressive symptoms reported more ruminative thoughts, but there were no sex differences in the relationship. With that being said, because women are more likely to ruminate and more likely to experience sexual violence, it is reasonable to assume that these ruminations may exacerbate the symptoms of depression, as previously suggested (68). Of course, not all ruminations are maladaptive and some degree of reflection is necessary in order to learn how to recover from trauma (77).

### Trauma-related thoughts and symptoms of anxiety and depression

**Figure 4:**
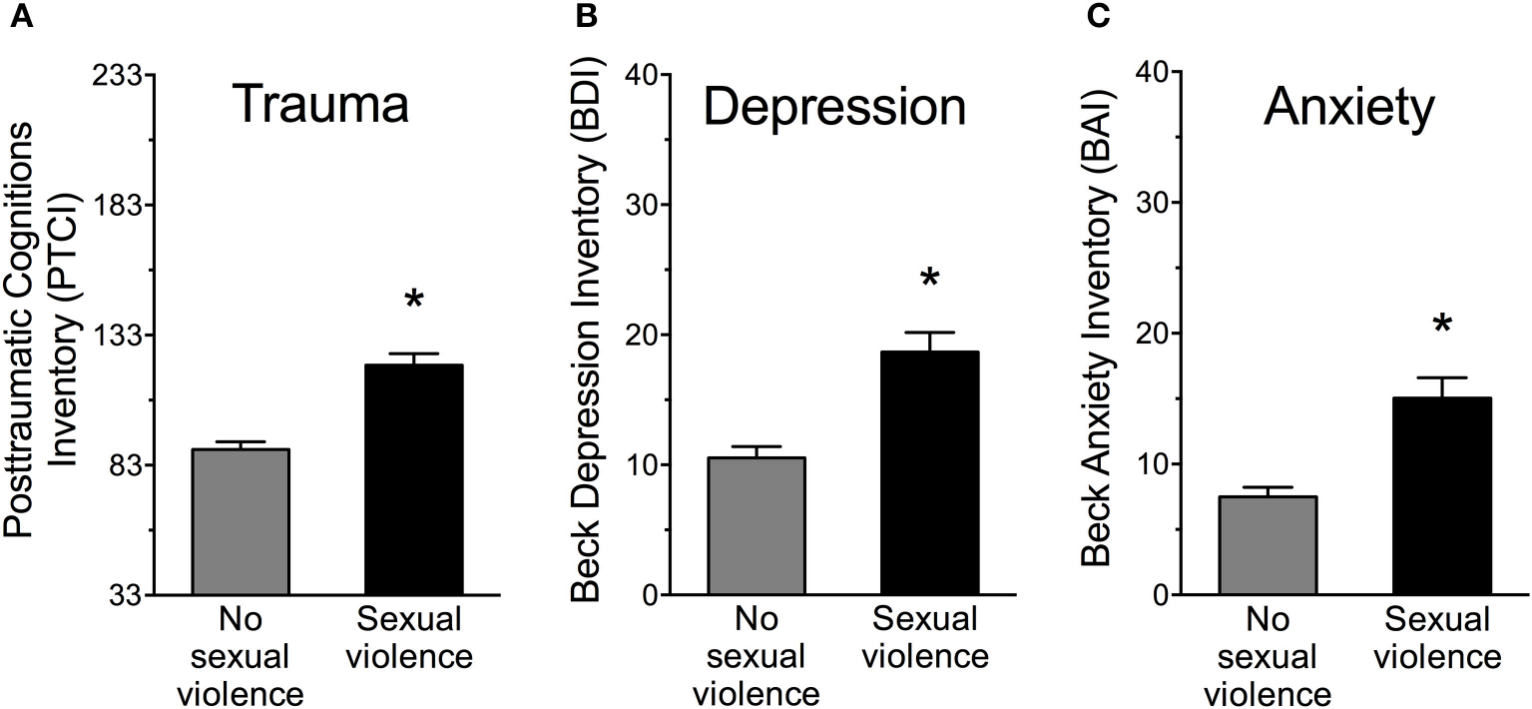
**(A)** Women with sexual violence history reported significantly more posttraumatic cognitions as assessed by the Posttraumatic Cognitions Inventory (PTCI), **(B)** more depressive symptoms as assessed by the Beck Depression Inventory (BDI), and **(C)** more anxiety symptoms as assessed by the Beck Anxiety Inventory (BAI) compared to women with no sexual violence history. ^*^p < 0.001.

Women with SV history had PTCI scores greater than 120, which is relatively high (Figure 4A), especially because most of them were not diagnosed with PTSD (16). However, participants without trauma history also had relatively high PTCI scores (16). These differences are easily explained. We asked participants to reflect on the most “stressful event” in their lifetime, rather than the most “traumatic event.” We did this in order to compare responses between participants with and without trauma history but obviously, this procedure would increase PTCI scores across groups. Importantly though, women with SV history reported more trauma-related thoughts than women without SV history. And women with SV and PTSD reported more posttraumatic thoughts than women with SV history and no PTSD.

Most women with SV history did not meet diagnostic criteria for a depressive or anxiety disorder, as diagnosed by the SCID. As a group, their BDI scores were consistent with a mild mood disturbance (39) and BAI scores were consistent with mild anxiety (40). Overall, these data indicate a significant yet moderate elevation of anxiety and depressive symptoms in response to SV history. Others report similar findings in college students with violence history (78, 79). These results likely reflect low level activation of the sympathetic nervous system and the hypothalamic-pituitary adrenal axis, both of which are associated with the persistent expression of symptoms of depression and anxiety (80–84).

### Sex differences

We concentrated on women and not men for several reasons. First, women are four times more likely to experience SV compared to men, and women between ages 12 and 34 are most likely to experience sexual assault (2, 6). Second, college-age women report sexual assault as the most traumatic event of their lifetime when compared to men (53% compared to 11%) (85). And third, women are more likely than men to develop assault-related and stress-related disorders such as PTSD, presumably because they are more frequently exposed to sexually violent events, but also perhaps due to biological differences in stress-related systems (86, 87).

The impact of SV on the human brain is difficult to study, in part because studies must be retrospective and variability between individual experiences is substantial (86, 88–90). To meet this need, we developed an animal model known as SCAR, which stands for Sexual Conspecific Aggressive Response. During this procedure, a young female rat in puberty is exposed to an adult sexually-experienced male each day for 30 min. As a result, female rats produced high levels of stress hormones and did not learn as well in standard laboratory training tasks. They also retained fewer new neurons in the hippocampus, a part of the brain necessary for many types of learning (91). These data suggest that the experience of sexual aggression can affect neuronal processes related to learning and memory, even neurogenesis. However, these results must be interpreted with caution; it is not possible to directly compare results from laboratory animal studies to women with SV history because the conditions surrounding the stressful events are different in many ways and levels of organization.

### Learning to recover

Sexual violence against women is an all too common occurrence and recent attention from the media is long overdue (1, 3). But this problem will not go away soon and we must keep our attention focused on prevention and justice for survivors. We must also find new ways to help women learn to recover. The most accepted course of treatment is Prolonged Exposure Therapy (PET) (12, 16, 24, 92), during which the client recollects the trauma memory during interviews, story-writing and even revisiting the traumatic location. After repeated exposure, the strength of the memory often lessens and the conditioned responses of fear and anxiety begin to extinguish (93, 94). Theoretically, the memory becomes less accessible in part because it has been updated and reconsolidated with other safer memories (95). Effective as it is, PET is time-consuming and can be expensive. Also, clients often drop out because it is emotionally painful to rehearse and relive the memory.

We recently developed a novel intervention to help women recover from the trauma of SV (26, 27, 96). The intervention is known as MAP Training because it combines “Mental And Physical” Training. Each session combines 30 min of mental training with silent meditation followed by 30 min of aerobic exercise. After 6 weeks of training, twice a week, women with SV history reported significantly fewer trauma-related cognitions and ruminative thoughts than women with SV history who were trained with meditation alone or exercise alone. Women who completed MAP Training also reported greater self-worth than the other training groups (26). It is unclear *how* this intervention works to reduce rumination and trauma-related thoughts. Women in the study completed the AMQ as well. Interestingly, scores did not change in women who completed training but increased in women who did not train. These data may suggest that fewer trauma-related and ruminative thoughts lessen the likelihood that the memory will strengthen over time.

## Conclusion

Women who experienced SV as adolescents or young adults reported intense memories for stressful life events, irrespective of PTSD. The strength of these memories was highly related to ruminative and trauma-related thoughts within individuals, as were symptoms of depression and anxiety. Theoretically, these data support the idea that ruminative thoughts and vivid memories of stressful life events coexist and are especially prominent in women who have experienced sexual violence. Thus, rumination may be an especially effective target for trauma recovery, because fewer ruminations would produce fewer vivid memories of trauma in the brain.

## Ethics statement

This study was carried out with written informed consent from all participants. IRB approval was obtained from Rutgers University for the study.

## Author contributions

TS, EM, and HC designed project. EM and HC carried out the study. TS, EM, and HC analyzed results. TS, EM, and HC wrote the manuscript.

### Conflict of interest statement

The authors declare that the research was conducted in the absence of any commercial or financial relationships that could be construed as a potential conflict of interest. The reviewer KL and handling Editor declared their shared affiliation.
